# Cognitive Behavioural Therapy to Optimize Post-Operative Fracture Recovery (COPE): protocol for a randomized controlled trial

**DOI:** 10.1186/s13063-022-06835-3

**Published:** 2022-10-22

**Authors:** Jason W. Busse, Sheila Sprague, The COPE Investigators, Gina Del Fabbro, Paula McKay, Lehana Thabane, Randi E. McCabe, Matilda Nowakowski, Christy Shibu, Natalie Fleming, Herman Johal, Gerard Slobogean, Roman M. Natoli, I. Leah Gitajn, Prism Schneider, P. J. Devereaux, Emil H. Schemitsch, Mohit Bhandari, Gordon H. Guyatt, Eleni Hapidou, Delia Chiaramonte, Henrick Kehlet, James Khan, Aaron Johnson, Diane Heels-Ansdell, Sofia Bzovsky, Brad A. Petrisor, Dale Williams, Bill Ristevski, Jamal Al-Asiri, Matthew Denkers, Kris Rajaratnam, Jodi L. Gallant, Sarah MacRae, Kaitlyn Pusztai, Sara Renaud, Nicki Johal, Steven Papp, Karl-Andre Lalonde, Bradley Meulenkamp, Allan Liew, Manisha Mistry, Braden Gammon, Wade Gofton, Geoffrey Wilkin, Melanie Dodd-Moher, David Puskas, Travis Marion, Tina Lefrancois, Jubin Payandeh, Claude Cullinan, Tracy Wilson, Kurt Droll, Michael Riediger, Rabail Siddiqui, Shalyn Littlefield, Simrun Chahal, Paige Wagar, Prism S. Schneider, Tosin Ogunleye, Tanya Cherppukaran, Karin Lienhard, Nicholas Smith, Sarah Anthony, Krista Butt, LaShann Selby, Murali Kovvur, Joshua Lawrence, Skyler Sampson, Kristin Turner, Todd Jaeblon, Haley K. Demyanovich, Sneh Talwar, Caroline Benzel, Theresa Chockbengboun, Devin Mullin, Logan Bateman, Melanie Christian, Peter DePalo, Paul J. Appleton, John J. Wixted, Edward K. Rodriguez, Michael F. McTague, Katiri Wagner, Kristina Brackpool, Kate Hegermiller, Nhi Nguyen, Courteney Fentz, Maricela Diaz, Jill Niceley, Kyle J. Jeray, Thomas M. Schaller, Michael S. Sridhar, John D. Adams, Richard W. Gurich, Stephanie L. Tanner, Kyle Adams, Michelle Donohue, Emily Bray, Calleigh Brignull, Harper Sprouse

**Affiliations:** 1 Department of Anesthesia, Michael G. DeGroote School of Medicine, McMaster University, HSC-2V9, 1280 Main St. West, Hamilton, L8S 4K1 Canada; 2 293 Wellington St. N, Suite 110, Hamilton, ON L8L 8E7 Canada

**Keywords:** Cognitive behavioural therapy, Persistent pain, Chronic pain, Orthopaedic, Trauma, Randomized controlled trial

## Abstract

**Importance:**

Chronic, non-cancer pain affects approximately 20–30% of the population in North America, Europe, and Australia, with surgery and trauma frequently cited as inciting events. Prospective studies of fracture patients have demonstrated an association between somatic pre-occupation, poor coping, and low recovery expectations following surgery with persistent pain, functional limitations, and lower rates of return to work. Psychological interventions, such as cognitive behavioural therapy (CBT), that are designed to modify unhelpful beliefs and behaviours have the potential to reduce persistent post-surgical pain and its associated effects among trauma patients.

**Objective:**

To determine whether online CBT, versus usual care, reduces the prevalence of moderate to severe persistent post-surgical pain among participants with an open or closed fracture of the appendicular skeleton.

**Design, setting, and participants:**

The Cognitive Behavioural Therapy to Optimize Post-Operative Fracture Recovery (COPE) protocol will be followed to conduct a multi-centre randomized controlled trial. Participants undergoing surgical repair of a long bone fracture will be randomized to receive either (1) online CBT modules with asynchronous therapist feedback or (2) usual care. The primary outcome will be the prevalence of moderate to severe persistent post-surgical pain over 12 months post-fracture. Secondary outcomes include the Short Form-36 Physical and Mental Component Summary scores, return to function, pain severity and pain interference over 12 months post-fracture, and the proportion of patients prescribed opioid class medications (and average dose) at 6 and 12 months post-fracture. The COPE trial will enroll 1000 participants with open and closed fractures of the appendicular skeleton from approximately 10 hospitals in North America.

**Discussion:**

If CBT is effective in improving outcomes among patients with traumatic fractures, our findings will promote a new model of care that incorporates psychological barriers to recovery.

**Trial registration:**

ClinicalTrials.gov Identifier: NCT04274530. Registered on 14 February 2020.

## Background

Chronic non-cancer pain affects 20 to 30% of the North American population, with similar prevalence in Europe and Australia [1–5]. Surgery and trauma are frequently cited as triggering events responsible for the development of chronic pain, a complaint that is associated with reduced quality of life and considerable socioeconomic burden. A UK survey of 5130 patients attending 10 outpatient pain clinics found that 41% attributed their chronic pain to a traumatic event or surgery, with 60% reporting chronic pain for > 2 years and 75% rating their pain as moderate or severe [6].

Stress, distress, anxiety, depression, catastrophizing, fear-avoidance behaviours, and poor coping strategies appear to be associated with both acute and chronic pain [7]. Previous studies of fracture patients have shown a strong association between somatic pre-occupation, poor coping, and low recovery expectations following surgery with persistent pain, functional limitations, and lower rates of return to work [8, 9]. Psychological interventions, such as cognitive behavioural therapy (CBT), that are designed to modify unhelpful beliefs and behaviours have the potential to reduce persistent post-surgical pain and its associated effects among trauma patients [10].

### Persistent pain among fracture patients

Clinical outcomes following operatively managed fractures of the extremities are variable and many patients continue to experience persistent pain and disability after surgery [11]. A prospective international cohort study of 14,831 patients that underwent non-cardiac surgery found that the highest prevalence of persistent pain at 12 months was associated with orthopedic surgery [12]. A 2006 systematic review of 20 observational studies of traumatic tibial fracture repairs found that 47% of patients experienced persistent post-surgical pain at an average of 24 months after surgery [13]. A subsequent clinical trial of 267 tibial fracture patients found 55% reported persistent post-surgical pain 12 months after surgery [8]. In another more recent trial involving 1560 patients with open extremity fractures, 67% of patients reported moderate to severe pain and 38% reported moderate to extreme pain interference at 12 months [11]. Several studies have found that psychosocial variables such as limited self-efficacy, poor social support, negative mood, and psychological distress are independent predictors for poor outcomes following surgery, including persistent pain, unemployment, and reduced physical function [14–17].

### Illness beliefs, persistent pain, and functional impairment

We previously developed and validated the Somatic Pre-Occupation and Coping (SPOC) questionnaire to explore the association between psychological factors and recovery following traumatic injuries. This 27-item questionnaire provides a total score that ranges from 0 to 162, with higher scores indicating worse coping, increased somatic complaints, lower energy, and pessimism regarding recovery [14]. The SPOC questionnaire has demonstrated high test–retest reliability (intra-class correlation coefficients for the total SPOC and all subscales range from 0.72 to 0.91), internal consistency (Cronbach’s *α* = 0.94), and construct validity [9]. Three separate prospective studies have found a strong association between higher SPOC scores in the acute post-operative period and greater risk of persistent pain, physical impairment, reduced quality of life, and unemployment at 12 months [9, 11, 14]. For example, among 1218 patients who underwent surgical repair for an open extremity fracture, those reporting SPOC scores of 74 or higher 6 weeks after surgery showed an increase in adjusted absolute risk of 41% for developing moderate to severe persistent pain, and an increase in adjusted absolute risk of 18% for moderate to severe physical impairment at 1 year following surgery.

A meta-analysis of 15 randomized controlled trials investigating the effectiveness of perioperative psychotherapy for a variety of surgical interventions found that patients who received active psychological interventions (CBT, relaxation strategies, or both) had significantly less persistent pain and physical impairment at 3 to 30 months follow-up compared to patients who received treatment as usual [10]. However, eligible trials were typically small (range = 15 to 120 patients) and the certainty in these treatment effects was downgraded due to imprecision and inconsistency.

### Need for a trial

Collectively, these findings suggest that psychological interventions designed to target unhelpful illness beliefs have the potential to improve prognosis in fracture patients. In response, we designed the Cognitive Behavioural Therapy to Optimize Post-Operative Fracture Recovery (COPE) trial to determine if an online CBT programme reduces persistent post-surgical pain and its associated effects in patients with operatively managed extremity fractures.

## Methods

### Protocol overview

The COPE trial is an exploratory, two-arm randomized controlled trial. This protocol outlines a multi-centre randomized controlled trial (RCT) evaluating the effectiveness of CBT delivered through an online platform for reducing persistent post-surgical pain and impairment in surgically managed extremity fracture patients. This trial will be coordinated by the Methods Centre within the Department of Surgery at McMaster University, Hamilton, Ontario, Canada. Methods Centre activities include trial oversight, clinical site management, data management, data analysis, and knowledge dissemination.

**Fig. 1:**
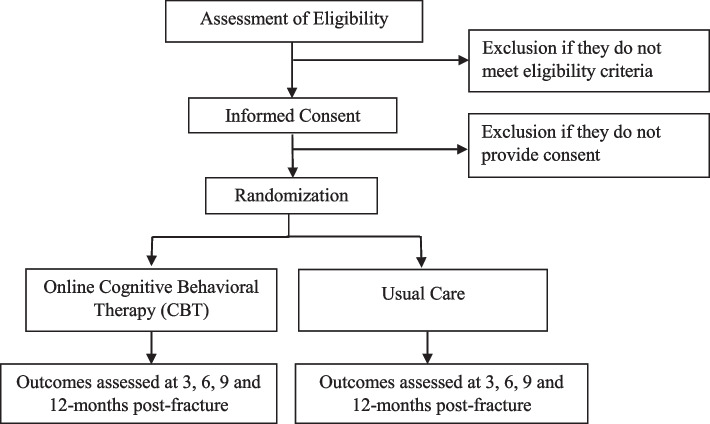
Overview of the COPE trial

The COPE trial is registered at ClinicalTrials.gov (NCT03673358), and our Methods Centre obtained ethics approval from Clinical Trials Ontario (CTO) (#2029) with the Hamilton Integrated Research Ethics Board (HiREB) as the Research Ethics Board (REB) of Record. This protocol is version 2.0, dated August 19, 2020. Each participating clinical site will also obtain ethics approval from either CTO or their local Institutional Review Board (IRB) or REB. Protocol modifications/amendments will first be submitted to CTO, and once approved, distributed to participating sites for local approval and updated at ClinicalTrials.gov. All participants will provide written informed consent prior to participating in any trial activities. An overview of the trial is provided in Fig. 1.

### Overarching objective and hypothesis

The primary objective of this trial is to determine if CBT, versus usual care, reduces the prevalence of moderate to severe persistent post-surgical pain over 12 months post-fracture in participants with an open or closed fracture of the appendicular skeleton. We hypothesize that the prevalence of persistent post-surgical pain over 12 months post-fracture will be lower in the CBT treatment group compared to the usual care group.

### Study setting, site eligibility, and selection of sites

This trial will be conducted across North America at approximately 10 academic hospitals (clinical sites) that manage patients with acute extremity fractures. To inform site evaluation and selection, all potential clinical sites will be asked to complete a site feasibility questionnaire evaluating (1) surgeon interest; (2) site research infrastructure, capacity, and resources; (3) estimates of key performance metrics including participant enrollment, follow-up, and compliance with the protocol based on their participation in previous trials. These questionnaires will be carefully reviewed by the Methods Centre prior to inviting a site to participate in the COPE trial. Throughout the trial, study sites may be added or removed based on performance (e.g., no enrolled patients in a consecutive 3-month period). A list of study sites can be obtained by contacting the corresponding author.

### Eligibility criteria

The following broad eligibility criteria will be used to increase generalizability of the trial:

1. Adult men or women aged 18 years and older.
2. Presenting to fracture clinic within 2–12 weeks following an acute open or closed fracture of the appendicular skeleton.
3. Fracture treated operatively with internal fixation.
4. Willing to participate in CBT.
5. Language skills and cognitive ability required to participate in CBT (in the judgement of site research personnel).
6. Consistent access to a smartphone and/or tablet that is capable of running the CBT provider’s application.
7. Provision of informed consent.

The exclusion criteria are:

1. Fragility fracture.
2. Stress fracture.
3. Concomitant injury which, in the opinion of the attending surgeon, is likely to impair function for as long as or longer than the patient’s extremity fracture.
4. Among patients who are fully weightbearing, those not experiencing any pain in the fracture region.
5. Active psychosis.
6. Active suicidality.
7. Active substance use disorder that, in the judgement of the treating surgeon, would interfere in the patient’s ability to partake in the CBT and/or the trial.
8. Already participating in, or planning to, start psychological treatments (including CBT) within the duration of the study (12 months).
9. Anticipated problems, in the judgement of study personnel, with the patient participating in CBT intervention and/or returning for follow-up.
10. Incarceration.
11. Currently enrolled in a study that does not permit co-enrolment in other trials.
12. Previously enrolled in the COPE trial.
13. Other reason to exclude the patient, as approved by the Methods Centre.

### Patient screening and consent

All patients 18 years or older with an open or closed fracture of the appendicular skeleton treated with internal fixation who present to a participating clinical site within 2–12 weeks (14–84 days) post-fracture will be screened for eligibility based on medical record review and discussions with the patient and their attending surgeon. A screening form will be completed for all patients who are assessed for trial eligibility (Fig. 1).

Patients who meet eligibility criteria will be invited to participate in the COPE trial. If it is not possible to discuss the study in person with the patient (e.g., physical distancing requirements due to COVID-19), a delegated member of the clinical care team may initiate the consent process by telephone, as approved by the REB of Record. Each participating site is responsible for adhering to an informed consent process that meets the requirements of their REB/IRB and Good Clinical Practice guidelines. Participants may withdraw consent at any time.

### CBT intervention

The intervention for the COPE trial is online CBT with asynchronous support from a licensed therapist. To maximize consistency, the CBT intervention will be centrally provided by a single commercial CBT provider, LifeWorks Inc., based on a CBT manual developed by members of our study team (RM, MN). The CBT manual was piloted with 2 patient partners that had recovered from a surgically managed appendicular fracture. Patient partners also provided feedback on the online CBT programme, including overall user experience and time for completion, as well as the clarity of the modules, instructions, and exercises.

Participants randomized to CBT will be encouraged to start treatment immediately. Participants will be given access to a therapist-guided, CBT programme delivered through an internet-enabled device (e.g., smartphone, tablet, laptop). The programme is accessed through AbilitiCBT (macortho.abiliticbt.com), a secure, online platform with content designed specifically for the COPE trial. LifeWorks Inc. will be responsible for ensuring all CBT therapists supporting the COPE trial are qualified through training and experience to deliver the CBT intervention in accordance with their standard procedures. All therapists are regulated health professionals or members of a professional college/association in the province/state in which they practice, and may be registered social workers, psychologists, psychotherapists, or counsellors. They will have a minimum Master’s level education and at least 2 years of post-Master’s clinical experience in providing CBT and are required to complete an intensive screening process with reference checks. The CBT intervention includes 7 online modules that focus on the following components:

1. Emotional processing of the experience of pain and introduction to the cognitive behavioural model;
2. Introduction to the biopsychosocial model of pain, cognitive strategies, behavioural strategies, mindfulness, and acceptance; and,
3. Optimizing functioning and preparing for the future.

**Fig. 2:**
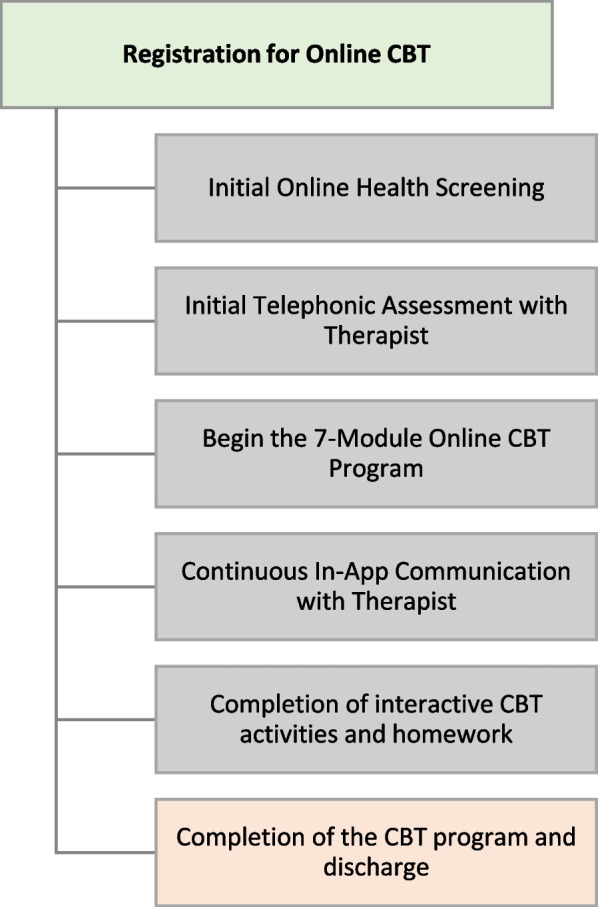
Process of the CBT intervention

CBT participants will complete an initial online health screening, providing their assigned therapist with information on their specific background and needs. The therapist will review the participant’s health screening, complete an initial telephonic assessment, and confirm the participant’s suitability for the programme. Participants will be provided with immediate access to modules 1 and 2. Each participant will progress through modules that consist of self-assessment tools, mental health screening tools, education material, videos, and CBT activities and homework (Fig. 2). The assigned therapist will help direct the programme, support goal development, monitor, encourage, and connect with the client through in-app messaging and telephone or video calling as appropriate throughout the programme. Subsequent modules are released upon completion of the previous module, required to only be opened in a standardized sequential format. Participants will also have 24/7 access to telephone crisis support.

In addition to therapy sessions, participants will be encouraged to complete homework to reinforce the application of CBT skills, between sessions. This is a key component of CBT, and participants will be encouraged to apply learned skills in their everyday lives. All CBT homework is completed and submitted directly through the mobile application and activity workbooks are reviewed by the therapist who will provide direction and support. In addition, CBT therapists will regularly meet with their clinical managers to discuss any learnings experienced throughout the programme and employ strategies to mitigate or overcome challenges.

In cases where participants disclose or show evidence of unstable behaviours (e.g., suicidal ideation, harm to self or others), LifeWorks Inc. will follow a risk assessment and mitigation process. High-risk cases are flagged in the case management system for priority outreach, completed by a crisis support counsellor. The crisis support counsellor completes a risk assessment before the participant begins their treatment and refers the participant to the most appropriate resource(s) or service. Once the risk assessment is completed, the crisis support counsellor maintains and determines the level of support required if the participant proceeds with treatment. LifeWorks Inc. therapists also follow a duty to report protocol should the participant communicate an explicit threat of physical harm or death to a clearly identified or identifiable victim(s). This may require the therapist to contact the appropriate authorities.

LifeWorks Inc. will provide the Methods Centre with reports summarizing participants’ compliance with CBT therapy and homework. This data will be used by the Methods Centre to provide weekly updates to participating sites regarding treatment compliance. Research Coordinators from each participating site will participate in regular online meetings with the Methods Centre to discuss learnings and challenges in order to optimize participant follow-up and treatment compliance. All participants will be followed for outcome collection regardless of compliance with treatment. All study participants will receive standard of care for their fracture by their treating surgeon.

### Blinding

Due to the nature of the intervention, it is not feasible to blind participants, study personnel, treating surgeons, or CBT therapists to treatment allocation. The data analyst(s) and study team members interpreting results will be blinded to treatment allocation. Once data interpretation has been finalized, the data will be unblinded, and the appropriate interpretation will be submitted for publication.

### Randomization

Participants will be allocated to either online CBT or usual care using the randomization function of the REDCap Cloud electronic data capture (EDC) system to ensure concealed allocation. Study personnel at each site will be responsible for randomizing eligible patients. Upon randomization, REDCap Cloud will assign each participant with a subject ID in sequence of enrollment at each site. Treatment allocation will be stratified based on the following factors to further promote prognostic balance between groups:

1. Clinical site (to account for systematic differences in perioperative care)
2. Sex
3. At least one open fracture versus no open fracture(s)
4. Military, veteran, or first responder vs. others
5. Greater illness beliefs (defined as SPOC score ≥ 48) versus lesser illness beliefs (SPOC score < 48).

### Primary and secondary objectives

The primary objective of our definitive trial is to determine if CBT, versus usual care, reduces the prevalence of moderate to severe persistent post-surgical pain over 12 months post-fracture in participants with an open or closed fracture of the appendicular skeleton. Our secondary objectives are to determine if CBT, versus usual care (1) increases physical functioning, (2) improves mental functioning, (3) accelerates return to function, (4) reduces pain severity, (5) reduces pain interference over 12 months post-fracture, and (6) reduces the proportion of participants prescribed opioid class medications (and average dose) at 6 and 12 months post-fracture. These outcomes will be assessed at 3 months, 6 months, 9 months, and 12 months post-fracture (Table 1).

**Table 1:** Schedule of Events for the COPE trial

**Schedule of events**	**Baseline visit** **2–12 weeks post-fracture** **(14–84 days)**	**CBT sessions** **(if applicable)** **(6–12 weeks post-fracture)**	3-month follow-up 2–4.5 months post-fracture(85–137 days)	6-month follow-up 4.5–7.5 months post-fracture(138–228 days)	**9-month follow-up** **7.5–11 months post-fracture** **(229–336 days)**	**12-month follow-up** **11–15 months post-fracture** **(337–456 days)**
**Assess eligibility**	X					
**Informed consent**	X					
**SPOC questionnaire**	X					
**Randomization**	X					
**Weightbearing and activity level form**	X					
**Demographic and baseline data**	X					
**CBT intervention**		X				
**Follow-up form**			X	X	X	X
**Persistent post-surgical pain assessment**			X	X	X	X
**BPI-SF Questionnaire**	X		X	X	X	X
**Return to Function Questionnaire**	X		X	X	X	X
**SF-36 Questionnaire**	X		X	X	X	X
**Fracture-related complications assessment**	X		X	X	X	X
**SAE assessment**			X	X	X	X

### Subgroup objectives and hypotheses

The COPE trial will explore for systematic differences in the effectiveness of CBT among a limited number of a priori subgroups, including (i) males versus females; (ii) any open fracture versus no open fracture; (iii) military, veteran, and first responders versus other patients; and (iv) higher versus lower SPOC scores (with greater illness beliefs defined as SPOC score ≥ 48 at baseline).

### Data collection and participant follow-up

Once participants have provided informed consent, study personnel will collect baseline demographics, relevant medical history, fracture characteristics, and surgical details from the participant and their medical records. Participants will complete the SPOC Questionnaire, SF-36, Return to Function questionnaire, and BPI-SF at the time of enrolment (Table 1).

Participants will be followed at standard clinical visit intervals for 12 months post-fracture and outcomes will be assessed at 3 months, 6 months, 9 months, and 12 months. At each follow-up visit, participants will complete the following questionnaires in the order specified below:

- Persistent Post-Surgical Pain (PPSP) Questionnaire
- Brief Pain Inventory-Short Form (BPI-SF) Questionnaire
- Return to Function Questionnaire
- SF-36 Questionnaire

At each follow-up visit, the following information will also be recorded: (1) fracture-related complications; (2) additional follow-up surgeries/procedures after the initial fracture surgery; (3) conversion to morphine equivalents for opioid class medications, and (4) serious adverse events (SAEs) potentially related to participation in the trial (Table 1).

The format of follow-up visits will be directed by participant’s preference (e.g., in-person at a scheduled clinic visit, or by a combination of telephone, email correspondence, text message, mail, alternate contact, and/or online via REDCap Cloud Survey). Questionnaires may be completed online directly by the participant or over the telephone with the assistance of research personnel. In addition to collecting follow-up data from the participant, clinical site personnel will review medical records to verify and/or supplement information.

**Fig. 3:**
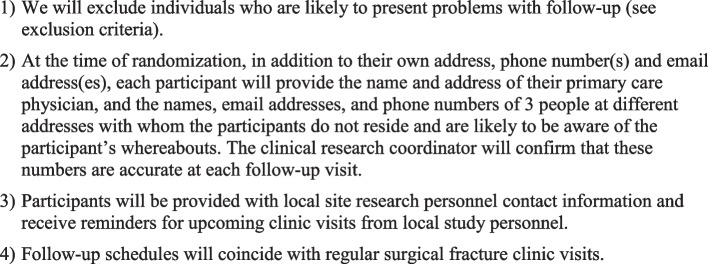
Retention strategies

Once a participant is enrolled in the trial, every reasonable effort will be made to follow the participant for the entire duration of the study period. The expected follow-up rate for this study is greater than 90% based on similar fracture trials conducted by our group [18–20]. Previously established procedures will be implemented to optimize participant retention (Fig. 3) [21, 22].

### Data management and data monitoring

Clinical sites will be provided with the COPE trial case report forms (CRFs) prior to the initiation of enrolment. Research personnel at each clinical site will submit the required data, as detailed on the CRFs, to the Methods Centre using the REDCap EDC system. Clinical site personnel will receive a unique login and password for the REDCap Cloud EDC system and will be able to view and modify data for participants recruited at their clinical site. The REDCap Cloud system uses a variety of mechanisms for checking data at the time of entry including skip logic, range checks, and data type checks. Upon receipt of new data, personnel at the Methods Centre will query all missing, implausible, or inconsistent data. Clinical site personnel will be notified of open queries through regular quality control reports and will be required to respond promptly. Due to the low-risk nature of the trial, a data monitoring committee will not be utilized for this trial.

LifeWorks Inc. will collect Personal Health Information (PHI) for participants randomized to the CBT intervention arm as required for their care. This PHI will be collected and stored in accordance with LifeWorks’ standard processes and procedures and will follow all applicable regulations including HIPAA and SOC 2 Type 1. LifeWorks Inc. will share treatment compliance data with the Methods Centre but will not share any other clinical data. All compliance data provided to the Methods Centre will be identified only by their assigned subject ID.

### Serious adverse events

Clinical sites are responsible for reporting SAEs that are potentially related to participation in the trial to the Methods Centre via the REDCap EDC system. To be potentially related to the trial, there must be reasonable cause to suggest that the CBT contributed to the adverse event. Significant new information on ongoing SAEs that are potentially related to participation in the trial will be provided promptly to the Methods Centre via the REDCap EDC system. Clinical sites are responsible for reporting SAEs to their local REB/IRB in accordance with local reporting requirements. The Methods Centre is responsible for submitting any SAEs to the REB of Record.

### Adjudication committee

The adjudication committee consists of two members, an orthopaedic surgeon, and a psychologist. This committee is responsible for rapid adjudication in cases where suspected ineligibility is identified by a clinical site or the Methods Centre. To be excluded from the trial after randomization, the participant must have been ineligible at the time of randomization. The adjudicators are provided with all relevant information, including any clinical/operative notes and case report forms to determine participant eligibility.

### Auditing

The Principal Investigators and Methods Centre personnel will monitor conduct at each clinical site over the course of the trial using the principles of risk-based monitoring. We will track key site performance metrics including screening and enrollment rates, quality and timeliness of data collection and entry, query resolution, and compliance with the protocol. The Principal Investigators and Methods Centre personnel will determine the need for, and timing of, ongoing monitoring visits based on these performance metrics. Monitoring visits will typically be conducted remotely but may also be conducted on-site at the discretion of the Principal Investigators. The Steering Committee is responsible for providing oversight for the COPE trial, and for providing recommendations on how to resolve any challenges that arise.

### Statistical plan

#### Sample size determination

The choice of sample size is based on a comparison of CBT versus usual care for the primary outcome of the prevalence of moderate to severe persistent post-surgical pain over 12 months post-fracture (Table 2). The anticipated rate of moderate to severe persistent pain is 67%, based on findings from 1218 patients with open extremity fractures enrolled in the FLOW trial, and followed for 1 year [11]. If we select a control group event rate of 50% and assume a 20% relative risk reduction with CBT, our required sample size per arm is 408. We will recruit 500 participants/arm, for a total of 1000 participants, to accommodate up to a 20% loss to follow-up.

**Table 2:** Sample size per arm (80% study power, alpha = 0.05). Difference in the proportion of persistent post-surgical pain, between treatment and control groups

		**Relative risk reduction in moderate to severe persistent pain**		
		15%	20%	25%
Proportion of moderate to severe persistent pain in control group (p1)	40%	1049	589	**376**
	45%	866	**488**	**313**
	50%	719	**408**	**262**
	55%	600	**342**	**221**

Table 2 shows the number of participants required per arm across a range of plausible baseline risks in the control group and risk differences of persistent post-surgical pain between treatment and control groups. Bolded numbers represent sample sizes/arm for which the trial is adequately powered to detect clinically important differences.

### Statistical analysis plan

A detailed statistical analysis plan (SAP) will be published prior to completion of the COPE trial. The analysis and reporting of results will follow the CONSORT guidelines for reporting of randomized controlled trials and will incorporate available updates to this guideline when available [23, 24]. The process of participant enrolment and engagement throughout the study will be summarized using a flow diagram. We will summarize participant demographics, fracture characteristics, fracture management details, and compliance with CBT, by treatment group, using descriptive summary measures, expressed as mean (standard deviation) or median (interquartile range) for continuous variables depending on the distribution, and frequency (percent) for categorical variables. We will also document the reasons for non-adherence with CBT among participants allocated to this treatment arm.

For all primary and secondary outcomes, participants will be analysed according to the treatment group to which they were allocated, regardless of the treatment they received (i.e., intention-to-treat). We will use logistic regression for analysis of binary outcomes and generalized estimating equations (GEE) assuming a serial auto-regressive (AR(1)) correlation error structure for continuous outcomes. For all models, the results will be expressed as effects (odds ratios for binary outcomes and mean difference for continuous outcomes) with corresponding two-sided 95% confidence intervals. An overview of the primary and secondary analyses is provided in Table 3.

**Table 3:** Primary and secondary analysis overview

**Objective**	**Outcome**		**Hypothesis**	**Method of Analysis**
	**Name**	**Type**		
To determine if CBT reduces the prevalence of moderate to severe persistent post-surgical pain over 12 months post-fracture	Persistent post-surgical pain as defined by the WHO, and of ≥ 4/10 severity	Binary	The prevalence of persistent post-surgical pain over 12 months post-fracture will be lower in the CBT treatment group compared to the usual care group	Logistic regression
Secondary Objective 1				
To determine if CBT improves physical and mental functioning over 12 months post-fracture	SF-36 PCS	Continuous	Participants receiving CBT will have higher SF-36 PCS scores over 12 months compared to participants who do not receive CBT	GEE
	SF-36 MCS	Continuous	Participants receiving CBT will have higher SF-36 MCS scores over 12 months compared to participants who do not receive CBT	GEE
Secondary Objective 2				
To determine if CBT improves return to function over 12 months post-fracture	Return to ≥ 80% of pre-injury functioning	Binary	The proportion of participants who report ≥ 80% of pre-injury functioning will be greater in the CBT group than the usual care group over 12 months post-fracture	Logistic regression
	Return to full function with respect to work, leisure activities, and responsibilities around the home	Binary	The proportion of participants who have returned, without limitations, to: (1) work, (2) leisure activities, and (3) responsibilities around the home will be higher among participants in the CBT group than participants in the usual care group	Logistic regression
Secondary Objective 3				
To determine if CBT reduces pain over 12 months post-fracture	BPI-SF Average Pain Severity Score	Continuous	Participants receiving CBT will have lower pain severity scores over 12 months compared to participants who do not receive CBT	GEE
	BPI-SF Pain Interference Score	Continuous	Participants receiving CBT will have lower pain interference scores over 12 months compared to participants who do not receive CBT	GEE
Secondary Objective 4				
To determine if CBT reduces the proportion of participants prescribed opioid class medications at 6 and 12 months	Taking an opioid class medication	Binary	The proportion of participants prescribed opioids at 6 and 12 months will be lower in participants receiving the CBT compared to participants who do not receive CBT	Logistic regression
	Amount of opioid class medication consumed	Continuous	Participants receiving CBT will be prescribed less opioids (morphine equivalent dose per day) compared to participants who do not receive CBT	GEE

We will also perform four a priori subgroup analyses as summarized in Table 4 and interpret the credibility of significant subgroup effects using ICEMAN criteria [25]. As the optimal methods for analysing data and presenting results from clinical trials continues to evolve, our statistical modeling techniques may be altered to reflect contemporary best practices at completion of participant follow-up.

**Table 4:** Subgroup analyses overview

**Objective**	**Outcome**		**Hypothesis**	**Method of analysis**
	**Name**	**Type**		
Subgroup Analysis 1				
Males versus females	PPSP as defined by the WHO, and of ≥ 4/10 severity	Binary	CBT will be associated with a larger reduction in the prevalence of PPSP in females compared to males	Logistic regression
Subgroup Analysis 2				
Any open fracture versus no open fracture	PPSP as defined by the WHO, and of ≥ 4/10 severity	Binary	CBT will be associated with a larger reduction in the prevalence of PPSP in participants with open fractures compared to participants with only closed fractures	Logistic regression
Subgroup Analysis 3				
Military, veteran, and first responders versus other patients	PPSP as defined by the WHO, and of ≥ 4/10 severity	Binary	CBT will be associated with a larger reduction in the prevalence of PPSP in participants who are employed by the military, veterans, or first responders	Logistic regression
Subgroup Analysis 4				
Higher versus lower SPOC scores	PPSP as defined by the WHO, and of ≥ 4/10 severity	Binary	CBT will be associated with a larger reduction in the prevalence of PPSP in participants with higher vs. lower SPOC scores	Logistic regression

*PPSP* Persistent post-surgical pain

Our primary analysis will consider only available data. If greater than 5% of data is missing, we will conduct a secondary analysis using multiple imputation. We plan to conduct two additional sensitivity analyses to explore the robustness of our findings. First, we will analyse different correlation structures for the error. Although the GEE method is robust to misclassification of correlation structure, we will re-examine the GEE analysis assuming an unstructured error structure to allow for an unequal number of participants within different treatment groups.

### Dissemination

The COPE Investigators will communicate the results of the trial to participants, healthcare professionals, and other relevant groups via a primary publication. Additional secondary papers may be published. We also plan to present the findings at relevant orthopaedic conferences across North America. The COPE Investigators consider the dissemination of results and data sharing to be important components of clinical research. Upon publication of the primary manuscript, a written proposal to the corresponding author will be required for the release of individual participant data and the data dictionary for the purposes of secondary analyses. Each request will need to include the research question of interest, planned methods, and specify which data points from the trial will be needed for the analysis. The COPE Principal Investigators will review each proposal and decide whether the secondary analysis is feasible. After a data sharing agreement is finalized, data files will be shared using McMaster University’s secure, online, cloud storage infrastructure.

## Discussion

Recovery from surgical fracture repair is highly variable, and there is increasing evidence that psychological factors, including unhelpful illness beliefs, are associated with long-term outcomes. To our knowledge, COPE will be the first large, multi-centre trial to evaluate the effect of online CBT with asynchronous therapist feedback on the development of persistent post-surgical pain and related outcomes among patients undergoing surgical repair for extremity fracture.

The COPE trial has several important strengths. We will enroll 1000 open and closed fracture patients from clinical sites across Canada and the USA, a sample size that ensures sufficient power to detect small but important differences. The CBT programme is widely accessible to participants, provided through any internet-enabled device (e.g., smartphone, tablet, laptop) in the state or province in which they reside, which will facilitate scalability if our trial is positive. We have implemented wide eligibility criteria to increase the generalizability of the trial, including an enrollment window of 2–12 weeks post-fracture and inclusion of any acute open or closed long bone fracture of the appendicular skeleton. In addition, we have established several methodological safeguards against bias: (1) employment of a variable block size with a centralized EDC system for randomization; (2) blinding of data analysts and manuscript writers to treatment allocation; (3) engagement of a centralized CBT provider to ensure consistency in treatment; (4) implementation of strategies to limit patients lost to follow-up; and (5) adjudication of patient eligibility when in doubt through an Adjudication Committee.

Persistent pain is common after fracture repair and is associated with poor coping and somatic pre-occupation after surgery. The COPE trial protocol provides a strong framework for the conduct of an RCT to determine the effectiveness of CBT in an orthopaedic trauma population. If CBT is effective in improving outcomes, our findings will promote a new model of care that considers psychological barriers to recovery after traumatic fracture repair.

## Trial status

This is version 2.0 of the COPE trial protocol, dated August 19, 2020. Recruitment for the trial began on December 14, 2020. Recruitment is expected to be completed in 2024.

## Data Availability

The Principal Investigators and research personnel from the McMaster Methods Centre will have access to the final trial dataset. This data may also be used for secondary publications, with permission from the Principal Investigators.

## Abbreviations

CBT: Cognitive behavioural therapy
COPE: Cognitive Behavioural Therapy to Optimize Post-Operative Fracture Recovery
SPOC: Somatic Pre-Occupation and Coping Questionnaire
RCT: Randomized controlled trial
CTO: Clinical Trials Ontario
REB: Research Ethics Board
IRB: Institutional Review Board
EDC: Electronic data capture
PPSP: Persistent post-surgical pain
BPI-SF: Brief Pain Inventory-Short Form
SAE: Serious adverse events
CRFs: Case report forms
PHI: Personal Health Information
GEE: Generalized estimating equation
SF-36 MCS: Short Form -36 Mental Component Summary
SF-36 PCS: Short Form -36 Physical Component Summary
WHO: World Health Organization
