# Correction: Cognitive Behavioural Therapy to Optimize Post-Operative Fracture Recovery (COPE): protocol for a randomized controlled trial

**DOI:** 10.1186/s13063-022-06889-3

**Published:** 2023-02-24

**Authors:** The COPE Investigators, Gina Del Fabbro, Paula McKay, Lehana Thabane, Randi E. McCabe, Matilda Nowakowski, Christy Shibu, Natalie Fleming, Herman Johal, Gerard Slobogean, Roman M. Natoli, I. Leah Gitajn, Prism Schneider, P. J. Devereaux, Emil H. Schemitsch, Mohit Bhandari, Gordon H. Guyatt, Eleni Hapidou, Delia Chiaramonte, Henrick Kehlet, James Khan, Aaron Johnson, Diane Heels-Ansdell, Sofia Bzovsky, Brad A. Petrisor, Dale Williams, Bill Ristevski, Jamal Al-Asiri, Matthew Denkers, Kris Rajaratnam, Jodi L. Gallant, Sarah MacRae, Kaitlyn Pusztai, Sara Renaud, Nicki Johal, Steven Papp, Karl-Andre Lalonde, Bradley Meulenkamp, Allan Liew, Manisha Mistry, Braden Gammon, Wade Gofton, Geoffrey Wilkin, Melanie Dodd-Moher, David Puskas, Travis Marion, Tina Lefrancois, Jubin Payandeh, Claude Cullinan, Tracy Wilson, Kurt Droll, Michael Riediger, Rabail Siddiqui, Shalyn Littlefield, Simrun Chahal, Paige Wagar, Prism S. Schneider, Tosin Ogunleye, Tanya Cherppukaran, Karin Lienhard, Nicholas Smith, Sarah Anthony, Krista Butt, La Shann Selby, Murali Kovvur, Joshua Lawrence, Skyler Sampson, Kristin Turner, Todd Jaeblon, Haley K. Demyanovich, Sneh Talwar, Caroline Benzel, Theresa Chockbengboun, Devin Mullin, Logan Bateman, Melanie Christian, Peter DePalo, Paul J. Appleton, John J. Wixted, Edward K. Rodriguez, Michael F. McTague, Katiri Wagner, Kristina Brackpool, Kate Hegermiller, Nhi Nguyen, Courteney Fentz, Maricela Diaz, Jill Niceley, Kyle J. Jeray, Thomas M. Schaller, Michael S. Sridhar, John D. Adams, Richard W. Gurich, Stephanie L. Tanner, Kyle Adams, Michelle Donohue, Emily Bray, Calleigh Brignull, Harper Sprouse

**Affiliations:** 1 Department of Anesthesia, Michael G. DeGroote School of Medicine, McMaster University, HSC-2V9, 1280 Main St. West, Hamilton, L8S 4K1 Canada; 2 Department of Surgery, McMaster University, 293 Wellington St. N, Suite 110, Hamilton, ON L8L 8E7 Canada

**Correction: Trials 23, 894 (2022)**

**https://doi.org/10.1186/s13063-022-06835-3**

Following publication of the original article [1], the authors identified an error in the authorship. Also, the 2^nd^ affiliation has been corrected.

The authorship and the 2^nd^ affiliation have been updated above and the original article has been corrected.
